# Impact of Pre-Treatment NLR and Other Hematologic Biomarkers on the Outcomes of Early-Stage Non-Small-Cell Lung Cancer Treated with Stereotactic Body Radiation Therapy

**DOI:** 10.3390/curroncol29010019

**Published:** 2022-01-04

**Authors:** Marina AduQuaye, Sheen Dube, Bashir Bashir, Amitava Chowdhury, Naseer Ahmed, Ahmet Leylek, Julian Kim, Pascal Lambert, Oliver Bucher, William Hunter, Gokulan Sivananthan, Rashmi Koul, Shrinivas Rathod

**Affiliations:** 1 Department of Radiation Oncology, CancerCare Manitoba, Winnipeg, MB R3E 0V9, Canada; maduquaye@cancercare.mb.ca (M.A.); dubes@learners.sjr.mb.ca (S.D.); bbashir1@cancercare.mb.ca (B.B.); achowdhury@cancercare.mb.ca (A.C.); nahmed2@cancercare.mb.ca (N.A.); aleylek@cancercare.mb.ca (A.L.); jkim7@cancercare.mb.ca (J.K.); whunter@cancercare.mb.ca (W.H.); gsivananthan@cancercare.mb.ca (G.S.); rkoul@cancercare.mb.ca (R.K.); 2 Department of Volunteer Services, CancerCare Manitoba, Winnipeg, MB R3E 0V9, Canada; 3 Department of Epidemiology and Cancer research, CancerCare Manitoba, Winnipeg, MB R3E 0V9, Canada; plambert@cancercare.mb.ca (P.L.); obucher@cancercare.mb.ca (O.B.); 4 Department of Radiation Oncology, Western Manitoba, Winnipeg, MB R7A 5M8, Canada

**Keywords:** biomarkers, early-stage lung cancer, stereotactic body radiation therapy

## Abstract

Introduction: We evaluated the association of pre-treatment immunologic biomarkers on the outcomes of early-stage non-small-cell lung cancer (NSCLC) patients treated with stereotactic body radiation therapy (SBRT). Materials and methods: In this retrospective study, all newly diagnosed early-stage NSCLC treated with SBRT between January 2010 and December 2017 were screened and included for further analysis. The pre-treatment neutrophil-lymphocyte ratio (NLR), monocyte lymphocyte ratio (MLR), and platelet-lymphocyte ratio (PLR) were calculated. Overall survival (OS) and recurrence-free survival (RFS) were estimated by Kaplan–Meier. Multivariable models were constructed to determine the impact of different biomarkers and the Akaike information criterion (AIC), index of adequacy, and scaled Brier scores were calculated. Results: A total of 72 patients were identified and 61 were included in final analysis. The median neutrophil count at baseline was 5.4 × 10^9^/L (IQR: 4.17–7.05 × 10^9^/L). Median lymphocyte count was 1.63 × 10^9^/L (IQR: 1.29–2.10 × 10^9^/L), median monocyte count was 0.65 × 10^9^/L (IQR: 0.54–0.83 × 10^9^/L), median platelet count was 260.0 × 10^9^/L (IQR: 211.0–302.0 × 10^9^/L). The median NLR was 3.42 (IQR: 2.38–5.04), median MLR was 0.39 (IQR: 0.31–0.53), and median PLR was 156.4 (IQR: 117.2–197.5). On multivariable regression a higher NLR was associated with worse OS (*p* = 0.01; HR-1.26; 95% CI 1.04–1.53). The delta AIC between the two multivariable models was 3.4, suggesting a moderate impact of NLR on OS. On multivariable analysis, higher NLR was associated with poor RFS (*p* = 0.001; NLR^1 HR 0.36; 0.17–0.78; NLR^2 HR-1.16; 95% CI 1.06–1.26) with a nonlinear relationship. The delta AIC between the two multivariable models was 16.2, suggesting a strong impact of NLR on RFS. In our cohort, MLR and PLR were not associated with RFS or OS in multivariable models. Conclusions: Our study suggests NLR, as a biomarker of systemic inflammation, is an independent prognostic factor for OS and RFS. The nonlinear relationship with RFS may indicate a suitable immunological environment is needed for optimal SBRT action and tumoricidal mechanisms. These findings require further validation in independent cohorts.

## 1. Introduction

Stereotactic body radiation therapy (SBRT) is an effective alternative treatment for patients with early-stage (stages IA, IB, or II) NSCLC who are medically inoperable or unwilling to undergo surgery, with cancer control and survival outcomes comparing favorably with those of surgical resection [1,2]. SBRT has been found to produce greater antitumor efficacy than would be predicted from standard radiological modelling alone, possibly through the superior engagement of the immune system, leading to enhanced antitumor immunity [3,4,5].

Markers of systemic inflammation—including circulating levels of neutrophils, monocytes, and lymphocytes—have been evaluated in the setting of different malignancies and have been found to predict response to therapy and disease outcomes. The neutrophil-to-lymphocyte ratio (NLR), platelet-to-lymphocyte ratio (PLR), and monocyte-lymphocyte ratio (MLR) are easily derived and inexpensive markers of systemic inflammation with prognostic value for survival in patients with various solid tumours [6,7,8,9,10].

Clinical outcomes after SBRT for early-stage NSCLC vary significantly between different studies: 3-year overall survival (OS) ranges from 37% to 72%, and recurrence rates vary from 18% to 29% rising the question about the selection of patients who will benefit from SBRT the most [11,12,13,14]. Utilizing prognostic factors—such as NLR, PLR, and MLR—obtained from complete blood count (CBC) could potentially inform decision-making in patients early-stage NSCLC considered for SBRT, given that it is readily obtained, minimally invasive and inexpensive. We, therefore, sought to determine whether pre-treatment immune biomarkers are predictive for cancer control and survival outcomes in patients with early-stage NSCLC managed with SBRT.

## 2. Materials and Methods

The local research ethics board approved this retrospective study. All newly diagnosed early-stage NSCLC (T1-2N0M0) patients treated with SBRT at CancerCare Manitoba between January 2010 and December 2017 were screened for inclusion and analysis. Patients with a previous history of malignancy apart from skin malignancy (excluding melanoma) were excluded from the study. Pre-treatment staging assessments included positron emission tomography (PET) and/or computed tomography (CT), chest-abdomen and cranial imaging (CT or MRI). Tissue diagnosis was preferred, and in cases where no tissue diagnosis was feasible, cases were discussed in local disease site group meetings for consensus on radiological diagnosis and treatment recommendations. In compliance with RTOG 0236 and RTOG 0815 trial protocols, tumours within 2 cm of the proximal bronchial tree were classified as central RTOG definition, and the rest were considered peripheral in location [2,15]. Peripheral lesions were treated with 48 Gy in 4 fractions, and central lesions were treated with a dose of 60 Gy in 8 fractions. Post-treatment patients were followed up with a CT scan of the chest at 3-, 6-, 12-, and 18-month post-treatment, and then every 12 months subsequently.

Patient-related characteristics were extracted manually from the electronic medical record including age, sex, Eastern Cooperative Oncology Group (ECOG) performance status score, forced expiratory volume in 1 s (FEV₁), diffusing capacity of carbone monoxide (DLCO), NLR pre-SBRT, PLR pre-SBRT, and MLR pre-SBRT. Tumour and treatment-related characteristics included T stage, maximum size (diameter), location, histology, and maximum standard uptake value (SUVmax) of positron emission tomography (PET), internal target volume (ITV) and planning target volume (PTV) and delivered doses. The overall survival (OS) interval was calculated from the date of radiation (first fraction of SBRT) to the date of death (any cause). Recurrence-free survival (RFS) was calculated from first fraction of SBRT to the time of radiological progression or last known follow-up date.

Cox hazard regression was used to assess the association of baseline variables with overall survival and recurrence-free survival. Univariable and multivariable analyses hazard regressions analysis were performed, including the following explanatory variables: NLR, PLR, MLR, ECOG performance status, ITV volume, age, and gender. Polynomial functions were used to account for any nonlinear relationships between predictors and outcomes. This was done because dichotomizing continuous predictors often reduces statistical power and variation between groups (e.g., individuals on either side of a cut point are seen as being very different) and does not indicate the possible nonlinear relationship [16,17]. Nonlinear relationships were presented using log (relative hazard) plots. To assess the impact of individual lymphocyte ratios, three metrics were computed: delta AIC, index of adequacy [18], and increase in integrated scaled Brier [18,19]. For this study, a delta AIC of 10 or more was considered to be a substantial improvement in model fit [20,21]. However, AIC values include sample size in their calculations. Therefore, delta AIC scores will increase in value for the same effect size as cohort size increases. The index of adequacy compares the likelihood ratio test of a model without the lymphocyte ratio to a model with the lymphocyte ratio. One minus the index adequacy indicates the fraction of new information provided by the lymphocyte ratio. The Brier score is the mean squared error and was scaled (1-(Brier/max Brier), where the max Brier is obtained from a Cox regression model without predictors. A scaled Brier score of 0 indicates a random association, and a value of 1 indicates perfect prediction. The proportional hazard assumption was tested using Schoenfeld residual plots. Kaplan–Meier survival plots of predicted values were produced with control variables held at their mean. Predictions were calculated for the 10th, 50th, and 90th percentile of each biomarker ratio.

## 3. Results

### 3.1. Patient Characteristics

In total, 72 ES-NSCLC patients were treated with SBRT from January 2010 to December 2017 at our institution, 10 of them were excluded from final analysis on account of missing CBC and one for missing ITV volume. Final cohort consisted of 61 patients included to final analysis. The median age of the cohort was 78 years with an interquartile range (IQR) of 72–82 years). Most of the patients were female (n = 41 (67.2%)), and 20 males (32.8%) were included. Eleven patients (18.0%) had an ECOG performance status 0, 37 patients (60.7%) had ECOG performance status 1–2, and in 13 patients (21.3%), ECOG performance status was unknown. Pretreatment histopathological diagnosis was available for 31 patients (50.8%), including 15 (24.6%) with adenocarcinoma, 10 (16.4%)–with squamous cell carcinoma, 5 (8.2%)–with NSCLC not otherwise specified, and one (1.6%) with biopsy positive for atypical cells not otherwise specified. Histopathology was unknown in 30 patients (49.2%).

The clinical tumour stage was T1a in 50 patients (82%), T1b in 10 patients (16.4%), and T2A in 1 patient (1.6%), respectively. The median tumour size was 2.1 cm with an IQR of 1.6–2.8 cm. The lesions were treated to a dose of 60Gy/8Fr in 9 patients (14.8%), 48Gy/4Fr in 50 patients (82%), and 60Gy/15Fr in two patients (3.3%). Eleven patients (18%) had central tumours. The median ITV and PTV volume were 11.2 and 35.6 cm³, respectively. In our cohort, 30 patients (49.1%) developed disease recurrence. The Median follow-up period was 2.14 years with the range of 0.1–5.6 years. Median OS duration was 3.0 years; 83.4% and 68.5% of patients were alive one year and two years after treatment, respectively. The main characteristics of the patients are illustrated in Table 1.

**Figure 1:**
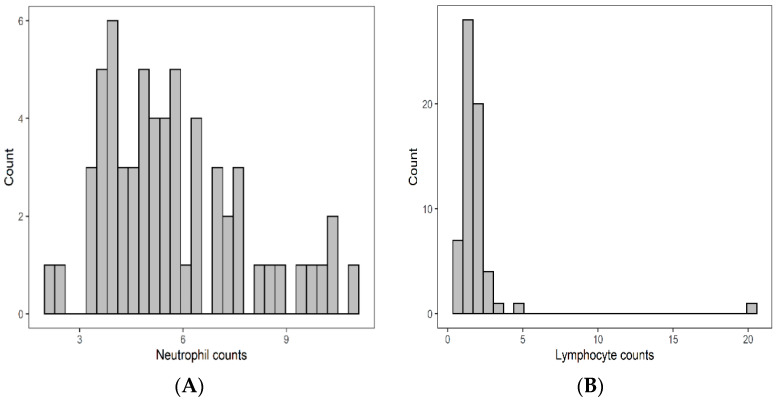

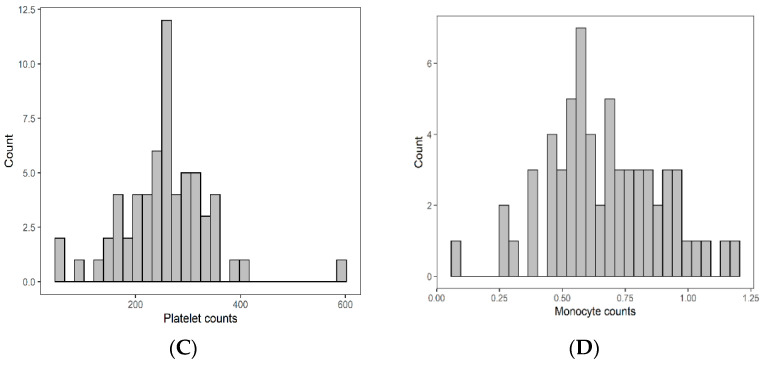
Pretreatment distribution of neutrophils (**A**), lymphcytes (**B**), monocytes (**C**), and platelet (**D**).

The baseline median neutrophil count at baseline was 5.40 × 10⁹/L (IQR: 4.17–7.05 × 10⁹/L), median lymphocyte count was 1.63 × 10⁹/L (IQR: 1.29–2.10 × 10⁹/L), median monocytes count was 0.65 × 10⁹/L (IQR: 0.54–0.83 × 10⁹/L) and median platelet count was 260.0 × 10⁹/L (IQR: 211.0–302.0 × 10⁹/L). Details are illustrated in Figure 1. Median NLR was 3.42 (IQR: 2.38–5.04), median MLR was 0.9 (IQR: 0.31–0.53), and median PLR was 156.4 (IQR: 117.2–197.5) respectively.

### 3.2. Survival Analysis

**Figure 2:**
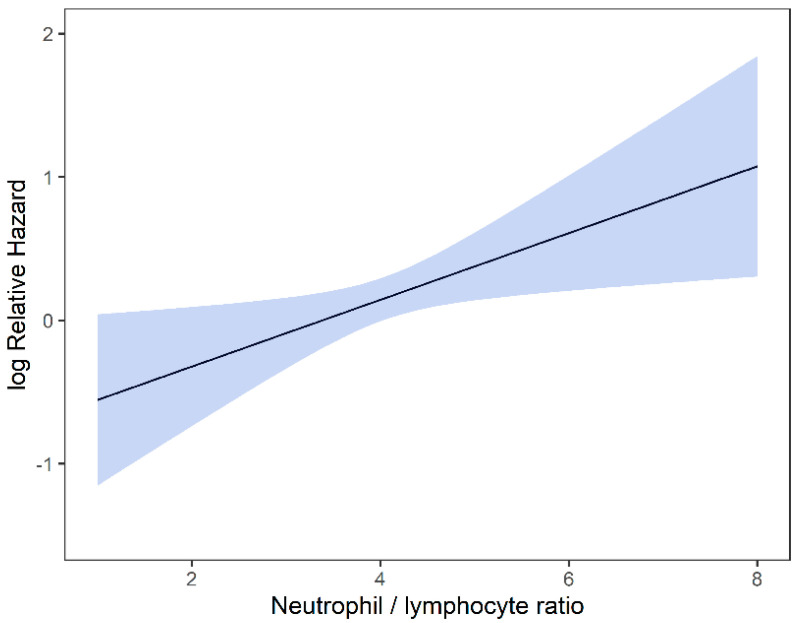
Risk of death by NLR.

**Figure 3:**
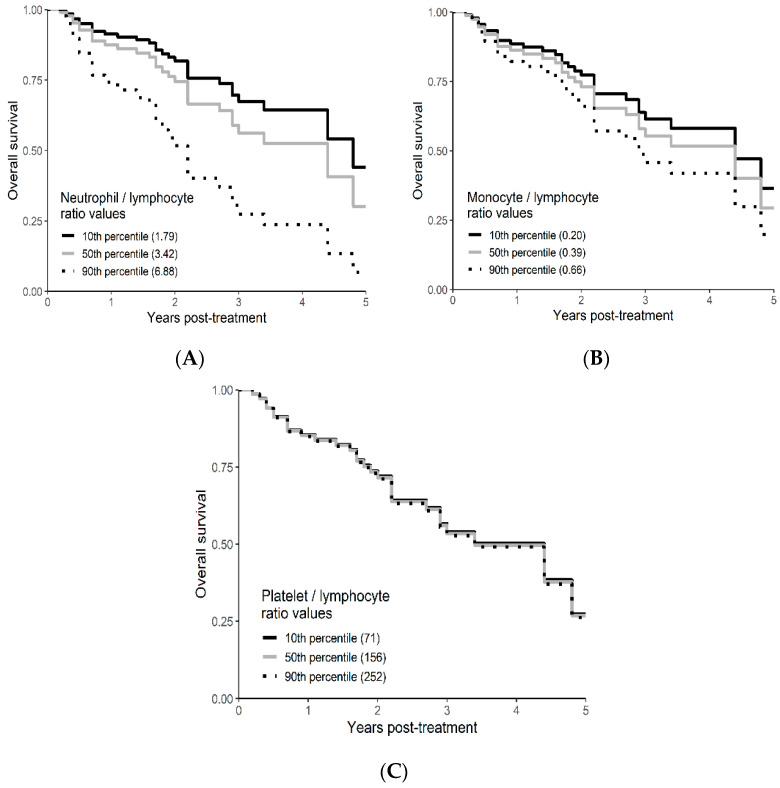
Kaplan–Meier curves for OS by NLR (**A**), MLR (**B**), and PLR (**C**) using predicted values at the 10th, 50th, and 90th percentile of predictors.

Univariable and multivariable analyses were performed for NLR, PLR, MLR, ECOG, ITV volume, age, and gender. On univariate analysis, higher NLR was associated with worse OS (*p* = 0.009; HR-1.27; 95% CI 1.06–1.53) and this relationship was linear (Figure 2). There was no association between PLR and OS (*p* = 0.833; HR-1.05; 95% CI 0.69–1.59). Similarly, MLR did not affect OS (*p* = 0.833; HR-2.92; 95% CI 0.62–13.78). Multivariable hazard regression models for overall survival including ECOG and ITV volume showed that higher NLR was associated with decreased OS (*p* = 0.017; HR-1.26; 95% CI 1.04–1.53), and the delta AIC between the two multivariable models was 3.4, suggesting a moderate impact on OS by NLR. The 1-index of adequacy was 0.52 and scaled integrated brier of 0.11. There was no association between MLR (*p* = 0.227; HR-2.80; 95% CI 0.53–14.86) and PLR (*p* = 0.930; HR-1.02; 95% CI 0.57–5.08) and OS; the delta AIC was less than 2, suggesting weak or no impact. Overall survival curves were calculated for 10th, 50th, and 90th percentile of NLR, MLR, and PLR are shown in figure (Figure 3), and demonstrates the larger impact of NLR on OS compared to MLR and PLR through the larger differences in OS estimates. The larger impact of NLR relative to MLR and PLR is also demonstrated with higher 1 minus index of adequacy values and scaled Brier increases (Table 2).

### 3.3. Recurrence Free Survival Analysis

**Figure 4:**
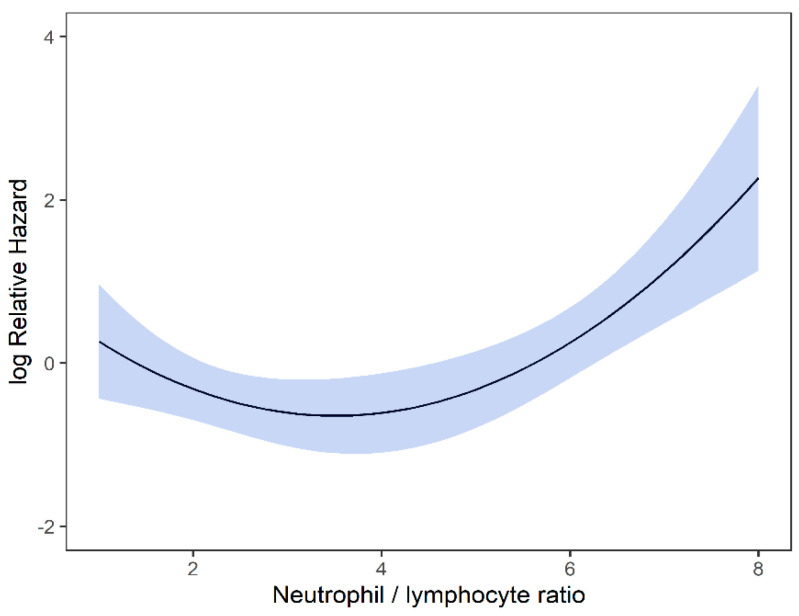
Risk of RFS by NLR.

**Figure 5:**
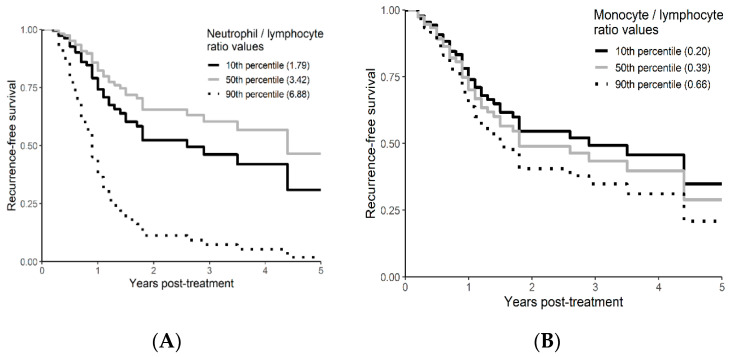

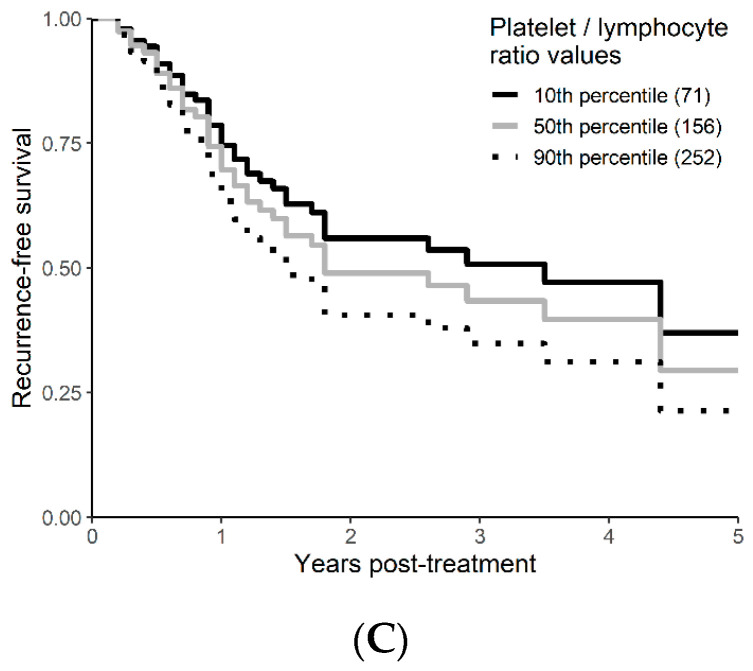
Kaplan–Meier curves for RFS by NLR (**A**), MLR (**B**), and PLR (**C**) using predicted values at the 10th, 50th, and 90th percentile of predictors.

On univariable analysis, higher NLR was associated with poor RFS (*p* = 0.01; NLR^1 HR-0.55; 95% CI 0.28–1.09; NLR^2 HR-1.8; 95% CI 1.01–1.17). The relationship between NLR and RFS was nonlinear, and a polynomial function was used (Figure 4). The best RFS was associated with NLR between 3.5–4.0 and the worst was above values 6. There was no statistically significant association between both MLR and PLR and RFS in univariate analysis (*p* = 0.340; HR-1.96; 95% CI 0.49–7.78 and *p* = 0.494; HR-1.13; 95% CI 0.80–1.60 respectively). On multivariable analysis, higher NLR was also associated with poor RFS (*p* = 0.001; NLR^1 HR-0.36; 95% CI 0.17–0.78; NLR^2 HR-1.16; 95% CI 1.06–1.26), and the delta AIC between two models was 16.20, implying a strong impact of NLR on RFS. The 1-index of adequacy was 0.7 and scaled integrated brier of 0.19. In our cohort MLR and PLR were not associated with RFS in multivariable models (*p* = 0.252; HR-2.35; 95% CI 0.54–10.43 and *p* = 0.241; HR-1.28; 95% CI0.85–1.92). For MLR and PLR, the delta AIC was less than 2, suggesting weak or no impact. Relapse-free survival curves were calculated based on the 10th, 50th, and 90th percentile of NLR, MLR, and PLR are shown in figure (Figure 5), which demonstrates the larger impact of NLR on RFS than MLR and PLR through larger differences in RFS estimates. The larger impact of NLR relative to MLR and PLR is also demonstrated with higher 1 minus index of adequacy values and scaled Brier increases (Table 3).

### 3.4. Local Recurrence Free Survival Analysis

**Figure 6:**
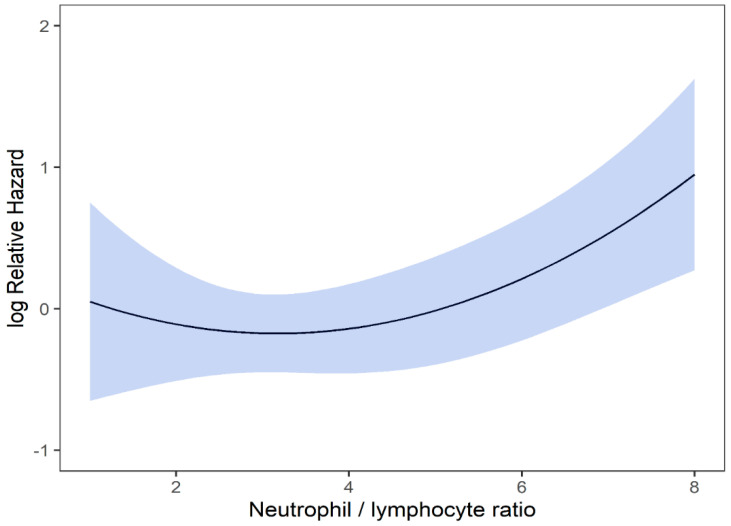
Risk of LRFS by NLR.

**Figure 7:**
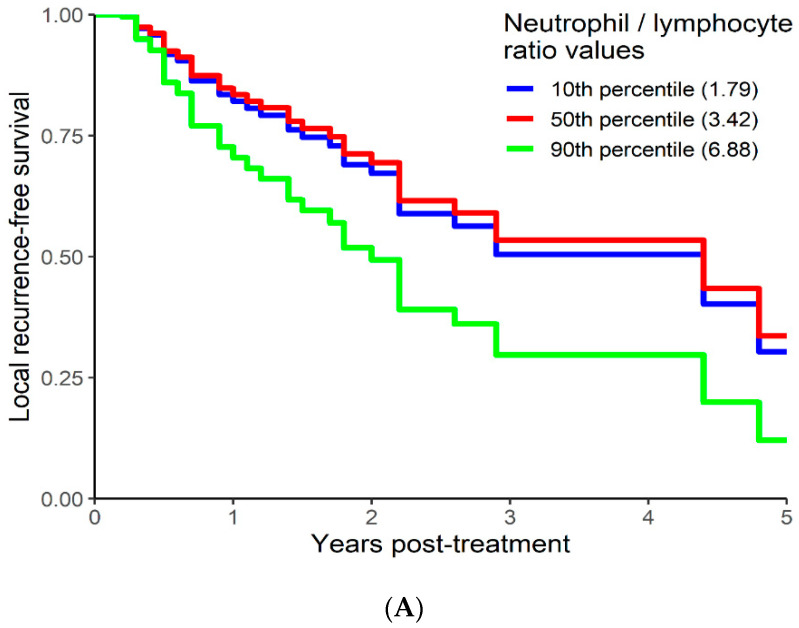

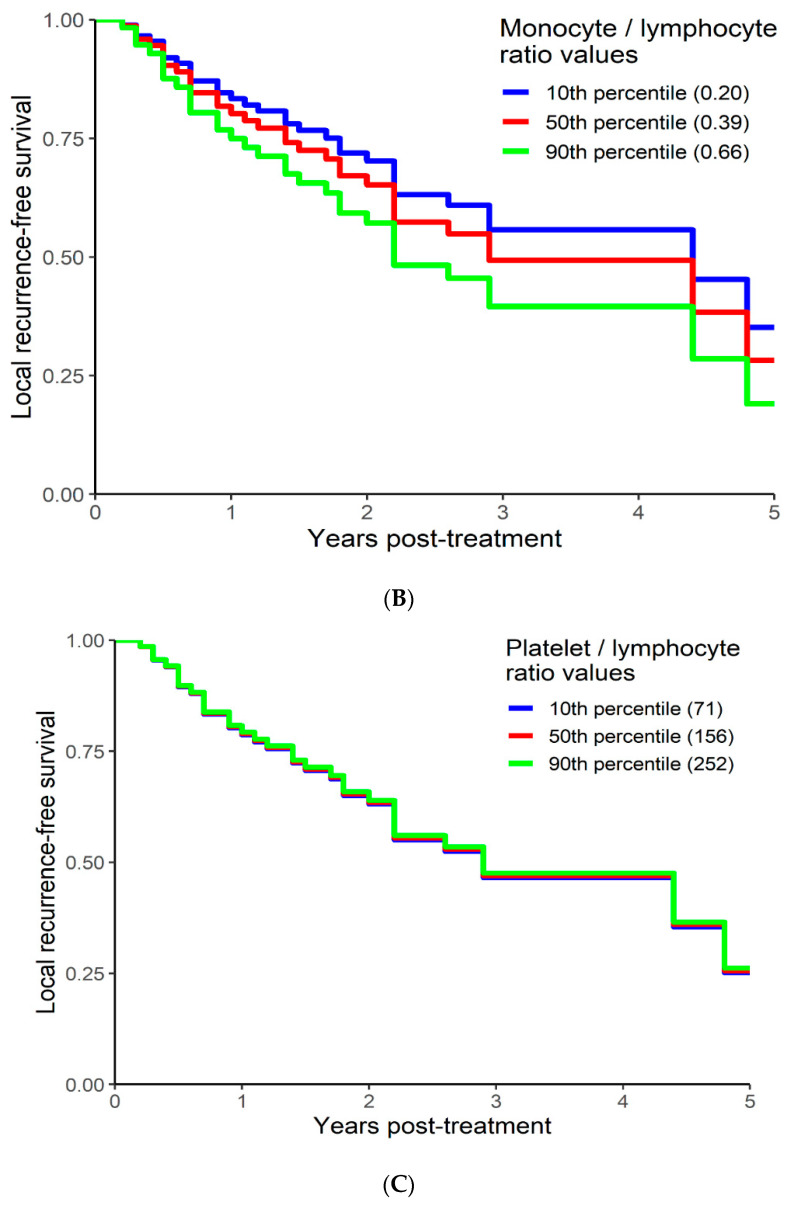
Kaplan–Meier curves for LRFS by NLR (**A**), MLR (**B**), and PLR (**C**) using predicted values at the 10th, 50th, and 90th percentile of predictors.

On univariable analysis higher NLR was associated with poor RFS (*p* = 0.01; NLR^1 HR-0.73; 95% CI 0.44–1.21; NLR^2 HR-1.05; 95% CI 1–1.1). The relationship between NLR and LRFS was nonlinear, and a polynomial function was used (Figure 6). The best LRFS was associated with NLR between 2.0–4.0 and the worst was above values 6. There was no statistically significant association between both MLR and PLR and LRFS in univariate analysis (*p* = 0.18; HR-2.71; 95% CI 0.63–11.69 and *p* = 0.958; HR-1.01; 95% CI 0.68–1.50 respectively). On multivariable analysis, higher NLR was also associated with poor LRFS (*p* = 0.021; NLR^1 HR-0.74; 95% CI 0.44–1.23; NLR^2 HR-1.05; 95% CI 1–1.1), and the delta AIC between two models was 4.09, implying a moderate impact of NLR on RFS. The 1-index of adequacy was 0.61 and scaled integrated brier of 0.14. In our cohort, MLR and PLR were not associated with LRFS in multivariable models (*p* = 0.212; HR-2.72; 95% CI 0.56–13.14 and *p* = 0.935; HR-0.98; 95% CI 0.66–1.47). For MLR and PLR, the delta AIC was less than 2, suggesting weak or no impact. LRFS survival curves were calculated based on the 10th, 50th, and 90th percentile of NLR, MLR, and PLR are shown in figure (Figure 7), which demonstrates the larger impact of NLR on LRFS than MLR and PLR through larger differences in LRFS estimates. The larger impact of NLR relative to MLR and PLR is also demonstrated with higher 1 minus index of adequacy values and scaled Brier increases (Table 4).

## 4. Discussion

Accumulated evidence shows plausible link between inflammation, particularly systemic inflammation, and cancer development and progression [7,8,9,10]. Systemic inflammation is known to promote tumour development and angiogenesis and inhibit apoptosis and has been reported to increase the risk of various cancers, such as liver, colorectal, breast, and lung cancer [7,8,9,10]. Neutrophil, platelet, lymphocyte, monocytes, and the ratios thereof could serve as a measure of inflammatory response and provide prognostic value in oncology [22].

The influence of surrogate markers for systemic inflammation on patient outcomes has been previously assessed and showed mixed findings [23,24,25]. Cannon et al. demonstrated that increased NLR and PLR were associated with poor overall survival with cutoffs of 2.98 and 146, respectively (*p* = 0.005 for NLR and *p* = 0.003 for PLR). However, when NLR and PLR were analyzed as continuous variables, they were not significantly associated with OS. Similarly, when NLR and PLR were analyzed as continuous variables, no significant association was found between nonlocal treatment failure and NLR (*p* = 0.937) and PLR (*p* = 0.133). Furthermore, no significant cutoff point was observed for NLR (AUC = 0.635; *p* = 0.15), but a PLR cutoff of 250 was found to maximize sensitivity and specificity (AUC = 0.720; *p* = 0.02) for nonlocal failure [23]. In a separate cohort, Shaverdian et al. found that higher pretreatment NLR and PLR independently predicted worse OS in early-stage NSCLC patients treated with SBRT on both univariate (*p* = 0.0003 and *p* < 0.0001 respectively) and multivariate analysis (HR-1.39; *p* = 0.0088 and HR-1.07; *p* = 0.024). The optimal NLR and PLR cutoffs in this study were 2.18 and 187.27, respectively. However, there was no correlation between NLR and PLR and locoregional (*p* = 0.81 and *p* = 0.25 respectively) or distant (*p* = 0.62 and *p* = 0.91 respectively) treatment failure [24]. Giuliani et al. demonstrated independent correlation between NLR (*P* < 0.01) and OS in early-stage NSCLC patients treated with SABR. Median OS was 4.3 years (95% CI 3.5 years to not reached) in patients with an NLR equal to or below the median (≤3, “low NLR”) and 2.5 years (95% CI 1.7 to 4.8 years) with NLR above the median (>3, “high NLR”). The correlation between MLR (*P* < 0.01) and disease-related failure was also found in this study [25]. The recent report with 389 patients showed although NLR was associated with OS, it was associated with non-lung cancer-specific survival and not lung cancer-specific survival [26].

In our study, multivariable models including ECOG and ITV volume showed that higher NLR was associated with decreased OS (*p* = 0.017; HR-1.26; 95% CI 1.04–1.53), and the delta AIC between the two multivariable models was 3.4, suggesting a moderate impact on OS. Our findings suggest an association between NLR with OS, and these would corroborate findings from previous reports [23,24,25]. We found that the higher pretreatment NLR values independently predicted poor OS.

In multivariable analysis, higher NLR was also associated with poor RFS (*p* = 0.001; NLR^1 HR-0.36; 95% CI 0.17–0.78; NLR^2 HR-1.16; 95% CI 1.06–1.26), and the delta AIC between two models was 16.20, implying a strong impact on RFS. In our cohort, MLR and PLR were not associated with RFS in multivariable models (*p* = 0.252; HR-2.35; 95% CI 0.54–10.43 and *p* = 0.241; HR-1.28; 95% CI0.85–1.92). On multivariable analysis, higher NLR was also associated with poor LRFS (*p* = 0.021; NLR^1 HR-0.74; 95% CI 0.44–1.23; NLR^2 HR-1.05; 95% CI 1–1.1), and the delta AIC between two models was 4.09, implying a moderate impact of NLR on RFS. The 1-index of adequacy was 0.61 and scaled integrated brier of 0.14. In our cohort MLR and PLR were not associated with LRFS in multivariable models (*p* = 0.212; HR-2.72; 95% CI 0.56–13.14 and *p* = 0.935; HR-0.98; 95% CI 0.66–1.47). In contrast to previous reports, our data suggest an NLR correlated with RFS and LRFS [23,24]. The previous studies reported cutoff values, and this may have decreased the sensitivity to detect relationship.

We did not use cutoff values for NLR, and our data demonstrated that, unlike for OS, the relationship of NLR with RFS and LRFS was nonlinear, with the risk of relapse increased when NLR values fell outside the optimal range. In our group of patients with early-stage NSCLC treated with SBRT had a lower RFS with NLR values between 3.5 and 4.5. Similarly, we found lower LRFS with NLR values between 2 and 4. To the best of our knowledge, this is the first study reporting the nonlinear relationship between NLR-RFS and NLR-LRFS. The nonlinear relationship may indicate an optimal immunological environment is needed for optimal SBRT action and tumoricidal mechanisms.

The conventional fractionated radiation therapy revolves around classical “4 Rs” including: repair, reassortment, reoxygenation, and repopulation. However, the radiobiology of SBRT is not fully explained by the 4Rs and resultant DNA damage, and additional mechanisms of vascular damage and antitumor immune response are also implicated [27]. Several reports suggest elevated immunomodulatory expression is associated with SBRT [27,28,29]. Understanding the role of systemic inflammation and its implications in SBRT is critical and of prognostic value. Thus, the utility and prognostic performance of systemic inflammation in stage I NSCLC undergoing SBRT are particularly interesting as the treatment mechanism.

Limitations: our study has several limitations and represents experience at our center, and a small number of patients of which (50.8%) had histopathological confirmation of the diagnosis. In addition, CBC draws from patients in this study were up to 3 months prior to initiation of SBRT, unlike other studies in surgical and chemotherapy series where CBC was done within 1 week to 1 month before treatment. It is unclear if the wider lead-time of CBC testing in our study had any material impact on the study results. Furthermore, the impact of baseline comorbidity (Charleson Comorbidity Index or other similar comorbidity indices) and immunomodulatory or anti-inflammatory medications could not be evaluated, and confounding could not be excluded. Therefore, our findings should be interpreted with caution and need confirmation in larger prospective studies.

## 5. Conclusions

Our study suggests NLR, as a marker of systemic inflammation, is an independent prognostic factor for worse OS, RFS, and LRFS. The nonlinear relationship with RFS and LRFS may indicate an optimal immunological environment is needed for optimal SBRT action and tumoricidal mechanisms. These findings require further validation in independent cohorts.

## Figures and Tables

**Table 1:** Patient demographic and characteristics.

Variable	Frequency
Patients (n)	61
Sex (n (%))	
Female	41 (67.2%)
Male	20 (32.8%)
Age (years)	
Mean (SD)	77.5 (6.6)
Median (Q1, Q3)	78 (72, 82)
Range	72–82
ECOG (n (%))	
0	11 (18.0%)
1–2	37 (60.7%)
missing	13 (21.3%)
ITV (cm³)	
Mean (SD)	18.0 (17.7)
Median (Q1, Q3)	11.2 (6.9, 20.8)
Range	1.10–94.80
PET SUV max (SD)	10.4 (6.2)
Histopathological diagnosis (total patients) (n (%))	32 (57.4%)
Adenocarcinoma	15 (24.6%)
Squamous cell carcinoma	10 (16.4%)
NSCLC	5 (8.2%)
Atypical cells	1 (1.6%)
Tumor size (cm)	
Mean (SD)	2.35 (1.0)
Median (Q1, Q3)	2.1 (1.6, 2.8)
Range	1.1–5.2
Clinical stage (n (%))	
T1A	50 (82%)
T1B	10 (16.4%)
T2A	1 (1.6%)
SBRT dose/fractions (n (%))	
60 Gy in 8 fractions	9 (14.8%)
48 Gy in 4 fractions	50 (82%)
60 Gy in 15 fractions	2 (3.3%)
Baseline neutrophils (×10⁹/L)	
Mean (SD)	5.75 (2.06)
Median (Q1, Q3)	5.40 (4.17, 7.05)
Range	2.07–10.89
Baseline lymphocytes (×10⁹/L)	
Mean (SD)	2.00 (2.46)
Median (Q1, Q3)	1.63 (1.29, 2.10)
Range	0.53–20.10
Baseline platelet (×10⁹/L)	
Mean (SD)	256.5 (85.9)
Median (Q1, Q3)	260.0 (211.0, 302.0)
Range	51.0–588.0
Baseline monocytes (×10⁹/L)	
Mean (SD)	0.67 (0.22)
Median (Q1, Q3)	0.65 (0.54, 0.83)
Range	0.08–1.19
Neutrophil/lymphocytes ratio	
Mean (SD)	3.91 (2.29)
Median (Q1, Q3)	3.42 (2.38, 5.04)
Range	0.27–13.69
Platelet/lymphocyte ratio	
Mean (SD)	169.9 (93.55)
Median (Q1, Q3)	156.4 (117.2, 197.5)
Range	146–534.6
Monocytes/lymphocytes ratio	
Mean (SD)	0.44 (0.22)
Median (Q1, Q3)	0.39 (0.31, 0.53)
Range	0.03–1
Death [n (%)]	29 (47.5%)
Recurrence [n (%)]	30 (49.1%)
Follow-up (years)	
Mean (SD)	2.28
Median	2.14
Median OS (years)	3.00

**Table 2:** Regression model for OS.

Variables		Univariable			Multivariable			Multivariable + Neutrophil/ Lymphocyte Ratio			Multivariable + Monocyte/ Lymphocyte Ratio			Multivariable + Platelet/ Lymphocyte Ratio		
		HR	95% CI	*p*	HR	95% CI	*p*	HR	95% CI	*p*	HR	95% CI	*p*	HR	95% CI	*p*
Neutrophil/lymphocyte ratio		1.27	1.06–1.53	0.009				1.26	1.04–1.53	0.017						
Monocyte/lymphocyte ratio		2.92	0.62–13.78	0.176							2.8	0.53–14.86	0.227			
Platelet/lymphocyte ratio	/100	1.05	0.69–1.59	0.833										1.02	0.66–1.57	0.93
ECOG	Missing	0.91	0.24–3.47	0.609	1.11	0.29–4.24	0.5049	1.29	0.32–5.13	0.3555	1.34	0.33–5.42	0.415	1.12	0.29–4.28	0.5031
	1–2	1.42	0.48–4.16		1.69	0.57–5.02		2.04	0.67–6.25		1.98	0.64–6.20		1.7	0.57–5.08	
	0	(ref)			(ref)			(ref)			(ref)			(ref)		
ITV (cm)	logged	1.44	0.99–2.10	0.06	1.47	1.01–2.15	0.045	1.38	0.93–2.05	0.114	1.44	0.98–2.13	0.064	1.47	1.01–2.15	0.046
Age	/10 years	1.13	0.64–2.01	0.664												
Gender	Female	0.94	0.43–2.03	0.873												
	Male	(ref)														
PET SUV max	/10	1.29	0.76–2.20	0.346												
AIC					203.3964			199.9933			204.0053			205.38888		
Delta AIC from multivariable								3.4031			−0.6089			−1.99248		
1 minus index of adequacy								0.52			0.22			0		
Scaled Integrated Brier					0.09			0.11			0.1			0.09		
Scaled Brier change (%)								17.4			1			−2.5		

OS—overall survival; ECOG—eastern cooperative oncology group; ITV—internal target volume; AIC—Akaike information criteria.

**Table 3:** Regression model for RFS.

Variables		Univariable			Multivariable			Multivariable + Neutrophil/Lymphocyte Ratio			Multivariable + Monocyte/ Lymphocyte Ratio			Multivariable + Platelet/ Lymphocyte Ratio		
		HR	95% CI	*p*	HR	95% CI	*p*	HR	95% CI	*p*	HR	95% CI	*p*	HR	95% CI	*p*
Neutrophil/lymphocyte ratio	^1	0.55	0.28–1.09	0.0098				0.36	0.17–0.78	0.0009						
	^2	1.08	1.01–1.17					1.16	1.06–1.26							
Monocyte/lymphocyte ratio		1.96	0.49–7.78	0.34							2.38	0.54–10.43	0.252			
Platelet/lymphocyte ratio	/100	1.13	0.80–1.60	0.494										1.28	0.85–1.92	0.241
ECOG	Missing	1.35	0.38–4.81	0.1387	1.57	0.44–5.62	0.0738	4.13	0.93–18.28	0.0052	1.86	0.49–6.99	0.0553	1.83	0.48–6.95	0.05
	1–2	2.45	0.86–7.02		2.97	1.02–8.58		9.24	2.25–37.90		3.4	1.13–10.28		3.6	1.14–11.42	
	0	(ref)			(ref)			(ref)			(ref)			(ref)		
ITV (cm)	logged	1.33	0.95–1.88	0.098	1.43	1.03–2.00	0.035	1.38	0.95–2.01	0.089	1.42	1.01–2.00	0.044	1.42	1.02–1.98	0.039
Age	/10 years	1.03	0.62–1.71	0.906												
Gender	Female	1.07	0.53–2.13	0.853												
	Male	(ref)														
PET SUV max	/10	1.21	0.75–1.95	0.443												
AIC					262.1701			245.9663			262.9462			262.8649		
Delta AIC from multivariable								16.2038			−0.7761			−0.6948		
1 minus index of adequacy								0.7			0.12			0.13		
Scaled integrated Brier					0.14			0.19			0.13			0.12		
Scaled Brier change (%)								31.5			−8.1			−18.3		

RFS—recurrence free survival; ECOG—eastern cooperative oncology group; ITV—internal target volume; AIC—Akaike information criteria.

**Table 4:** Regression model for LRFS.

Variables		Univariable			Multivariable			Multivariable + Neutrophil/ Lymphocyte Ratio			Multivariable + Monocyte/ Lymphocyte Ratio			Multivariable + Platelet/ Lymphocyte Ratio		
		HR	95% CI	*p*	HR	95% CI	*p*	HR	95% CI	*p*	HR	95% CI	*p*	HR	95% CI	*p*
Neutrophil/lymphocyte ratio	^1	0.73	0.44–1.21	0.001				0.74	0.44–1.23	0.021						
	^2	1.05	1.00–1.10					1.05	1.00–1.10							
Monocyte/lymphocyte ratio		2.71	0.63–11.69	0.180							2.72	0.56–13.14	0.212			
Platelet/lymphocyte ratio	/100	1.01	0.68–1.50	0.958										0.98	0.66–1.47	0.935
ECOG	Missing	0.90	0.24–3.40	0.407	1.06	0.28–4.03	0.311	1.27	0.33–4.95	0.239	1.27	0.32–5.11	0.256	1.05	0.28–4.03	0.314
	1–2	1.59	0.55–4.62		1.89	0.64–5.57		2.22	0.74–6.66		2.20	0.71–6.78		1.88	0.64–5.57	
	0	(ref)			(ref)			(ref)			(ref)			(ref)		
ITV (cm)	logged	1.37	0.93–2	0.110	1.42	0.97–2.09	0.074	1.30	0.86–1.97	0.217	1.39	0.93–2.07	0.105	1.43	0.97–2.10	0.074
Age	/10 years	1.22	0.71–2.11	0.477												
Gender	Female	1.10	0.52–2.32	0.813												
	Male	(ref)														
PET SUV max	/10	1.40	0.82–2.38	0.215												
AIC					227.69			223.60			228.23			229.69		
Delta AIC from multivariable								4.09			−0.54			−2.00		
1 minus index of adequacy								0.61			0.22			0.00		

LRFS—local recurrence free survival; ECOG—eastern cooperative oncology group; ITV—internal target volume; AIC—Akaike information criteria.

## Data Availability

The data presented in this study could be shared in deidentified format on specific request, as per local institutional policy. Such request should be directed to the corresponding author.
